# The Exocrine Pancreas in Cystic Fibrosis in the Era of CFTR Modulation: A Mini Review

**DOI:** 10.3389/fped.2022.914790

**Published:** 2022-06-27

**Authors:** Isabelle R. McKay, Chee Y. Ooi

**Affiliations:** ^1^Wagga Wagga Base Hospital, Wagga Wagga, NSW, Australia; ^2^School of Clinical Medicine, Discipline of Paediatrics and Child Health, Randwick Clinical Campus, University of New South Wales (UNSW) Medicine and Health, University of New South Wales, Sydney, NSW, Australia; ^3^Department of Gastroenterology, Sydney Children's Hospital Randwick, Randwick, NSW, Australia

**Keywords:** cystic fibrosis, exocrine pancreas, CFTR modulators, pancreatitis, precision medicine

## Abstract

Cystic fibrosis (CF) is a common disorder of autosomal recessive inheritance, that once conferred a life expectancy of only a few months. Over recent years, significant advances have been made to CF therapeutic approaches, changing the face of the disease, and facilitating the partial restoration of pancreatic function. This mini review summarizes the current landscape of exocrine pancreatic management in CF and explores areas for future direction and development.

## Introduction

Cystic fibrosis (CF) is one of the most common recessive genetic disorders worldwide, with wide-ranging health implications. While CF is characterized as an illness of pulmonary morbidity and mortality, it is a multisystem disorder encompassing sequelae in the gastrointestinal, hepatobiliary, and pancreatic systems (1). CF occurs due to mutations in the cystic fibrosis transmembrane conductance regulator (CFTR) gene. Over 2,000 mutations, with varying functional consequences, have been identified to date, resulting in a spectrum of disease phenotypes. The CF gene encodes the CFTR protein responsible for driving chloride, bicarbonate and fluid secretion in affected epithelial surfaces. Dysfunction in the CFTR protein results in thick, inspissated secretions which in turn leads to obstruction, infection, inflammation and ultimately destruction of affected organs (2). These processes result in clinical manifestations such as chronic sinopulmonary diseases, exocrine and endocrine pancreatic diseases, intestinal obstruction, cirrhosis and obstructive azoospermia from atrophic or absent vasa deferens (3). This mini-review will focus on the exocrine pancreatic disease in CF, and how presentation and management has evolved with the introduction of CFTR-modulating therapies.

## Pathophysiology of Cystic Fibrosis in the Pancreas

Pancreatic dysfunction in CF is a result of ductal obstruction from early in life. The specific mechanisms behind this process are multifactorial and incompletely understood, but are thought to hinge on dysregulated bicarbonate buffering, altered chloride flux and the production of pro-inflammatory pancreatic secretions. Our understanding of the pathophysiology has evolved exponentially in recent years, and is continuing to expand rapidly. The first pathological descriptions of “fibrocystic disease” of the pancreas were published by Dr. Dorothy Andersen in 1938 (4), but it was not until 1989, almost four decades later, that the CFTR gene was first recognized by Riordan and colleagues, demonstrating the clear genetic basis for the multisystem phenotype of CF (5).

In the 30 years since, our understanding of the role of CFTR in pancreatic function has deepened, guiding CF management beyond a “blanket” approach and toward more directed therapy. Today, CFTR has been established as a key modulator of chloride and bicarbonate transport, with downstream implications for several organ systems. In the small pancreatic ducts, this ionic flux is crucial in the production of alkaline fluid, the neutralization of gastric acid and the maintenance of a functional environment for digestive enzymes (2).

In the pancreatic ductal epithelia, defective CFTR results in ductal secretions with a lower pH (secondary to reduced bicarbonate buffering), a lower volume (secondary to reduced sodium chloride-driven osmosis) and an increased viscosity (secondary to protein hyperconcentration) (6, 7). This process begins *in utero*, resulting in early acinar plugs consisting mainly of zymogen material. As exocrine atrophy progresses, the ability of the pancreas to produce zymogens decreases, and these plugs come to contain a higher proportion of mucins secondary to ductal metaplasia (8). Ultimately, the end effects of CFTR dysregulation have been widely observed to result in pancreatic ductal obstruction and zymogen accumulation, leading to fibroinflammatory changes and parenchymal injury (2, 9).

## Genotype-Phenotype Correlations of the Exocrine Pancreas in CF

The spectrum of phenotypic presentation of exocrine pancreatic disease in CF closely correlates with individual genotype. While >2,000 mutations have been identified to date, with more expected to be uncovered with time, they can be broadly categorized into six classes in relation to the degree of CFTR protein production and function. These classes are summarized in Table 1 below.

**Table 1:** An overview of the genotype classes in CF, with their associated pancreatic phenotype (11–13).

**Class**	**CFTR function**	**Pancreatic status**	**Example Mutations**
I “*Protein synthesis defect”*	No CFTR is synthesized due to stop codons or splicing defects.	Insufficient	G542X, W1282X, R553X, 3950delT
II “*Maturation defect”*	CFTR is synthesized but in an immature form which is degraded intracellularly.	Insufficient	F508del, N1303K
III “*Gating defect”*	Despite synthesis of CFTR, activation and regulation by ATP or cAMP are disrupted.	Insufficient	G551D, G178R, S549N, S549R, G551S, G970R, G1244E, S1251N, S1255P, G1349D
IV “*Conductance defect”*	CFTR is synthesized and expressed at the plasma membrane, but chloride conductance is reduced.	Insufficient	R334W, G314E, R347P, D1152H
V “*Reduced quantity”*	CFTR synthesis is normal but produced in quantities too small to be effective at the cell surface.	Sufficient	3849+ 10 kb C→ T, 3272-26 A→ G, 2789+5G→ A
VI “*Reduced stability”*	CFTR stability is reduced so protein synthesis at the cell surface cannot occur in quantities high enough to be effective.	Variable	c. 120del123, rPhe580del
Unclassified			All other mutations, including those unknown

Genotype classes I, II and III are commonly associated with pancreatic insufficiency, due to a greater degree of CFTR deficiency and dysfunction. Genotype classes IV, V, and VI or compound heterozygous individuals with one less severe allele, tend to retain some level of exocrine function as CFTR is still produced to some extent (Table 1). Response to therapeutic interventions varies between genotype classes, and varies further within each class itself. While genotype provides one method of classifying CF disease to guide investigation and management, it is important to recognize that outcomes in CF are also influenced by a number of other determinants including epigenetic factors, genetic modifiers, environmental factors and socioeconomic status (11).

## CFTR Modulator Therapy Overview

The identification of CFTR as the genetic basis for CF disease has turned scientific research toward precision medicine, and the early 2000s saw the introduction of CFTR modulator drugs. CFTR modulators are designed to support or restore the functionality of CFTR, and are classified into five key groups based on mechanism: potentiators, correctors, stabilizers, read-through agents, and amplifiers (3). Currently, only four agents have been made available on the pharmaceutical market, and are approved for use only in specific genotypes.

### Potentiators

Potentiators restore or augment the cAMP mediated CFTR gating, allowing for some degree of CFTR-dependent transport to occur. Approximately 5% of CF mutations are a gating or conductance deficit, and it is this proportion of the CF population that will benefit from potentiator therapy. These mutations tend to fall within Classes III and IV (Table 1). Currently, the only available potentiator is ivacaftor, which is approved use either as a monotherapy, or as combination therapy with the correctors tezacaftor or lumacaftor. While the mechanism of action is yet to be fully elucidated, clinical outcomes demonstrate significant improvement amongst eligible patients commenced on therapy, ranging from decreased frequency of pulmonary exacerbations, to nutritional improvement and rescued pancreatic function (14, 15). While there are several other potentiators currently under clinical evaluation, ivacaftor remains the only potentiator available for patient use.

### Correctors

Correctors (e.g., tezacaftor, lumacaftor, and elexacaftor) assist CFTR protein structuring and trafficking. While the specifics of activity vary between individual agents, the mechanism of the corrector class is considered to be either direct (binding to the misfolded protein itself and “chaperoning” it through the endoplasmic reticulum) or indirect (through proteostasis regulation) (3). Mistrafficking is the most common mutation type in CF, notably including the F508del mutation.

### Stabilizers

Stabilizing therapies target Class VI mutations, wherein the CFTR protein is present at the plasma membrane but has reduced availability due to increased lysosomal degradation. Stabilizers function to anchor the protein at the cell surface, preventing premature removal and destruction. While lumacaftor has been shown to transiently increase CFTR stability, ongoing investigation into agents with longer-term benefits are ongoing (16).

### Read-Through Agents

One of the more severe phenotypes in CF results from defective CFTR synthesis in the first instance, usually due to the introduction of a premature termination codon (PTC) into the protein mRNA (Class I mutations). Read-through agents allow the protein translation process to “skip over” the PTC through recruiting an alternative amino acid in its place, facilitating the production of a full-length protein (17). Aminoglycosides such as gentamicin have demonstrated read-through capabilities in early experimental studies, but the practical application of these properties is limited by the toxicity profile seen with longer-term dosing. While some trials are currently examining aminoglycoside derivatives with stronger read-through capability and less toxicity, nothing has been approved for use in CF to date (18, 19).

### Amplifiers

Class V mutations encompass those genotypes resulting in reduced synthesis or maturation of the CFTR protein. Amplifiers target this class of mutation, increasing the expression of CFTR mRNA and the downstream protein production load (20). Nesolicaftor is currently in the midst of phase 3 trials (Proteostasis Therapeutics), having shown promising results throughout earlier studies with significantly higher CFTR mRNA levels when used in combination with existing corrector and potentiator therapies (21, 22).

## The Exocrine Pancreatic Phenotypes in CF

Two key clinical manifestations present themselves as hallmarks of exocrine pancreatic disease in CF: (1) pancreatic insufficiency, and (2) symptomatic pancreatitis among a subset of people with pancreatic sufficient (PS) CF. Each of these confer a distinct disease burden and occur within a specific subset of the CF population, shaping presentation and management.

### Pancreatic Insufficiency

Approximately 85% of people with CF are pancreatic insufficient (PI) from early in life (23). A near absolute loss in exocrine output results in a disease of maldigestion, characterized by steatorrhoea, failure to thrive, and fat-soluble vitamin deficiencies. Left unrecognized, this population historically died in early infancy from malnutrition, before the now-familiar pulmonary manifestations of CF even took root (24). Under- or malnutrition (as measured by BMI) has been shown in epidemiological studies to be closely linked with poor pulmonary and survival outcomes in CF (25). Nowadays, the appropriate and timely initiation of pancreatic enzyme replacement therapy (PERT), coupled with a high energy diet, allows these individuals to facilitate nutritional digestion and maintain adequate growth and development; hence early recognition and management are essential. Even with PERT treatment, the clinical consequences of PI are ongoing and continues to represent a large proportion of CF morbidity and mortality, leading to malnutrition, poor weight gain and a decreased ability to withstand intercurrent clinical insults (26).

The PI status should always be confirmed on testing. Fecal elastase is the most commonly utilized test in clinical practice for assessing exocrine pancreatic function. Traditionally, a fecal elastase cutoff of <200 μg/g as indicative of PI is used although a lower cutoff of 100 μg/g has been reported to be of greater predictive value for ruling out PI and minimize false positive results due to dilution of feces caused by non-pancreatic intestinal causes (e.g., short gut syndrome) (27). Other indirect measures of exocrine pancreatic function include fecal chymotrypsin and serum trypsinogen. These latter two tests have a lower sensitivity and specificity than fecal elastase, and their use in the diagnostic setting is curbed by some key clinical limitations. Fecal chymotrypsin is a commercially available enzyme, so it cannot be reliably measured in patients prescribed PERT (28). Serum trypsinogen is not specific for exocrine pancreatic function, and can be elevated in other states of disease including acute pancreatitis and is not widely available (29). Direct tests of pancreatic function, such as secretin or cholecystokinin testing (using a dreiling tube or endoscope), are more sensitive and specific, but less commonly used due to their technical nature and poor patient tolerance and/or need for general anesthesia (in children) (30).

### CFTR-Related Pancreatitis

**Figure 1:**
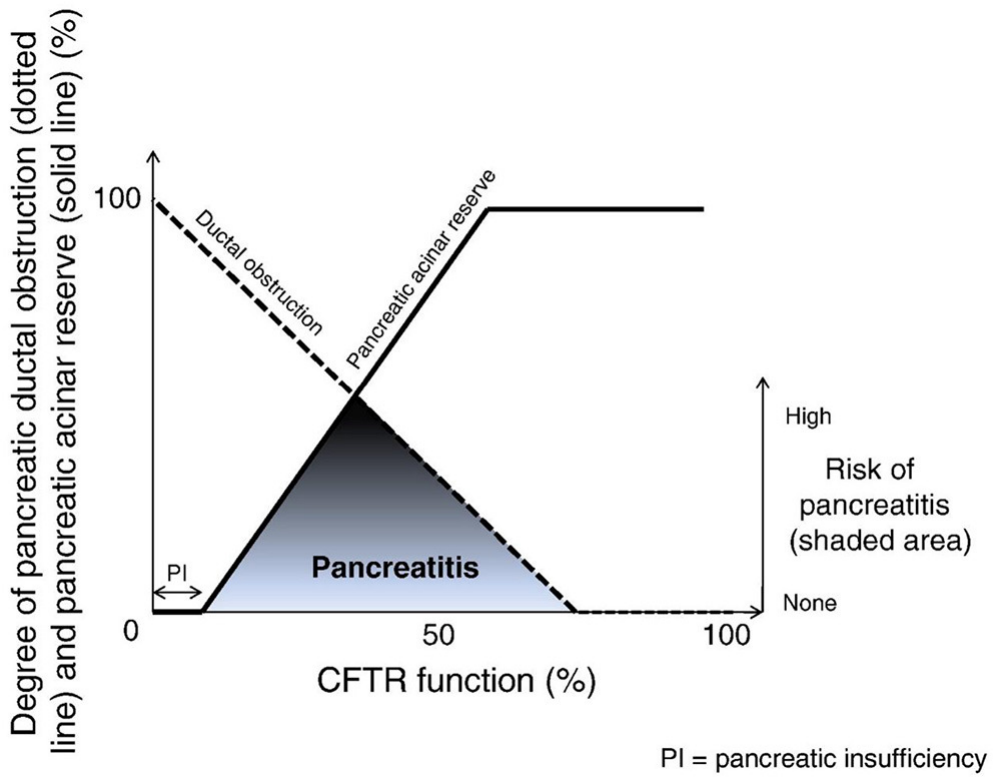
The development of pancreatitis in the context of CFTR-related contributors. Adapted from Ooi et al. (10).

Approximately 15% of the CF cohort are PS, born with at least 2% of residual pancreatic reserve, enough to adequately digest and absorb nutrients (26). While PS individuals tend to exhibit a less severe phenotype overall, it is in this population that symptomatic pancreatitis may occur, at a proportion of ~20%. It is thought to result from an altered ratio of acinar reserve and ductal obstruction, as conceptualized in Figure 1. Compounding this process, impaired bicarbonate secretion leads to altered luminal pH, promoting ongoing acute tissue inflammation and the perpetuation of pancreatitis in the chronic period (31). This pathology relies on a degree of acinar function to be preserved in the presence of significant ductal obstruction, hence a negligible prevalence of pancreatitis amongst the PI group.

As well as genotype being a key determinant, risk of pancreatitis is also amplified in the context of cigarette or alcohol use, both of which independently decrease CFTR function (32, 33). Pancreatitis in the CF cohort is diagnosed with classical criteria of archetypal symptomatology, serum amylase/lipase greater than three times the upper limit of normal, and/or consistent findings on imaging (34).

It is also worth noting that in the context of the progressive nature of pancreatic disease in CF, a percentage of PS individuals will become PI later in life. The likelihood of this level of pancreatic destruction is largely informed by genotype (e.g., carriage of classes I, II or III mutations on both alleles) and previous bouts of symptomatic pancreatitis (10). This suggests that recurrent attacks of symptomatic pancreatitis heralds progressive deterioration in exocrine function, underscoring the importance of serially monitoring function amongst the PS group.

## Three Distinct CFTR Modulator Treatment Groups in CF Pancreatic Disease

CFTR modulator treatment eligibility and planning is generally based on genotype classing (as described in Table 1). However, this patient classification system does not incorporate likely pancreatic response into the decision-making process. As CFTR modulator use has increased, longitudinal evidence comes forth, describing three different patient groups with distinct pancreatic presentations and response to therapy.

### Pancreatic Insufficient Individuals May Recover Pancreatic Function

Pancreatic dysfunction in CF begins *in utero*, and while PERT prescription can assist in mitigating some of the clinical ramifications, exocrine disease persists throughout life (35). As CFTR modulators have become established as a key tenet of CF management, their wide-ranging effects on health beyond pulmonary capacity have been brought to the fore. Emerging evidence has demonstrated improvement and recovery in exocrine pancreatic function, with implication for growth and nutritional outcomes later in life. The ARRIVAL trial noted improvements in measured pancreatic markers such as fecal elastase (FE) and immunoreactive trypsinogen (IRT) when ivacaftor was introduced in young children between 12 and 24 months (36), and the KIWI/KLIMB studies demonstrated similar improvements amongst a group in early childhood (aged 2–5 years) (37, 38). However, the magnitude of these benefits is curbed as the introduction age of ivacaftor increases, and ferret models have demonstrated that withdrawal of therapy reinstates exocrine disease (39, 40). Ultimately, these findings support the concept that the “window of opportunity” to rescue exocrine pancreatic function in CF occurs early in life, hinging on early and sustained modulator therapy.

The “window of opportunity” in initiating CFTR modulator treatment is likely to be from as early in life as possible. However, a recent ivacaftor study in ferret models demonstrated partial protection from disease progression when the therapy was commenced *in utero*. Ferrets in the treatment group demonstrated similar growth rates as wild-type animals, and continued on a normal growth trajectory while nursing even without PERT treatment (40). Corroborating this proof-of-concept, a recent human case report provides details of a child born to a F508del homozygous mother on elexacaftor/tezacaftor/ivacaftor treatment. Despite inheriting a F508del homozygous genotype themselves, this child did not meet the laboratory criteria for the IRT CF newborn screening, and was born PS with growth tracking along the 85th centile (41). Together, these studies support the finding that pancreatic disease begins *in utero*, and raises the possibility that modulator treatment commenced even prior to birth may slow or perhaps prevent exocrine disease from taking hold. Currently, no modulator therapy is approved for use earlier than 2 years of age.

### Individuals With Recovered Pancreatic Function May Develop Symptomatic Pancreatitis

Rescuing exocrine pancreatic function in CF has clear benefits for nutrition and growth. However, increasing pancreatic reserve in CF poses its own risks, as a growing body of evidence highlights links to the development of symptomatic acute pancreatitis amongst a subset of patients who were PI prior to commencement of modulators, corroborated by the findings of recent case reports. Gould et al. describe a series of five patients, all PI, who developed a classical presentation of acute pancreatitis at a median of 30 months following commencement of modulator therapy. Of these five, three had regained some level of exocrine function with FE measurements above 100 μg/g (42). Megalaa et al. describe a similar case report of a 10 year old child, realized to have regained PS status only after an episode of acute pancreatitis (43). In keeping with the model conceptualized by Ooi and colleagues in 2011 (Figure 1), this suggests that while CFTR modulators may effectively increase pancreatic acinar reserve, in the setting of ongoing ductal obstruction, this can result in a heightened risk of pancreatitis (10). This speaks to the risk profile of CFTR modulators, highlighting that their health benefits must be considered in the context of potential serious complications which clinicians must remain vigilant for amongst this population.

### Pancreatic Sufficient Individuals May Have a Reduced Risk of Symptomatic Pancreatitis

Conversely, PS patients commenced on modulator treatment appear to accelerate beyond this critical ratio of acinar reserve and ductal obstruction (Figure 1), with a subsequent decline in the prevalence of pancreatitis amongst this group. Akshintala et al. retrospectively reviewed a small cohort of adult CF patients with a history of pancreatitis in the preceding 2 years, highlighting that none of these 15 individuals developed pancreatitis during their follow up period (mean 36 months) (44). Ramsey and colleagues supported these findings 3 years onwards, demonstrating a significant reduction in pancreatitis-related hospitalizations amongst those commenced on CFTR modulators amongst both PI and PS patients, with a greater relative risk reduction within the PS group (45). Overall, these findings suggest that in those suffering from recurrent pancreatitis, or who have a pre-existing risk of pancreatitis, CFTR modulators may assist in shifting away from this “risk window,” alleviating ductal obstruction enough to improve pancreatic output without inducing further inflammation.

## Conclusion

Pancreatic disease represents a significant proportion of CF-related morbidity and mortality. Where treatment previously focused mitigating the effects of downstream sequalae, the research landscape has shifted now to focus on addressing the central CFTR mutation at the root. As CFTR modulators become established as a cornerstone of CF management, the limitations of these novel agents are brought to the fore. The risk-benefit profile of these therapies varies for three CF cohort subsets, depending on pre-existing pancreatic function and risk of pancreatitis. This classification of modulator-eligible patients encourages a patient-centered treatment approach, where a distinct risk monitoring process may facilitate the early recognition of key complications unique to each population. The temporal outcomes of CFTR modulator use in the context of longer-term pancreatic complications including CF-related diabetes and malignancy are yet to be established, but with demonstrable benefits in the short-term setting, positive effects may be anticipated.

## Author Contributions

IM and CO conceptualized the topic and drafted review design. IM analyzed the literature and drafted the manuscript. CO refined the manuscript and provided guidance on literature interpretation. All authors made significant and direct contributions to the work and approve it for publication.

## Conflict of Interest

CO has served as a consultant and received research funding (unrelated to the content of this manuscript) from Vertex Pharmaceuticals. The remaining author declares that the research was conducted in the absence of any commercial or financial relationships that could be construed as a potential conflict of interest.

## Publisher's Note

All claims expressed in this article are solely those of the authors and do not necessarily represent those of their affiliated organizations, or those of the publisher, the editors and the reviewers. Any product that may be evaluated in this article, or claim that may be made by its manufacturer, is not guaranteed or endorsed by the publisher.
